# Temperature Stability of the Sky Quality Meter

**DOI:** 10.3390/s130912166

**Published:** 2013-09-11

**Authors:** Sabrina Schnitt, Thomas Ruhtz, Jürgen Fischer, Franz Hölker, Christopher C.M. Kyba

**Affiliations:** 1 Institute for Space Sciences, Freie Universität Berlin, Carl-Heinrich-Becker-Weg 6-10, Berlin 12165, Germany; E-Mails: Sabrina.schnitt@fu-berlin.de (S.S.); ruhtz@zedat.fu-berlin.de (T.R.); juergen.fischer@wew.fu-berlin.de (J.F.); 2 Leibniz-Institute of Freshwater Ecology and Inland Fisheries, Müggelseedamm 310, Berlin 12587, Germany; E-Mail: hoelker@igb-berlin.de

**Keywords:** light pollution, sky quality meter, light at night, temperature, stability, skyglow, night sky brightness

## Abstract

The stability of radiance measurements taken by the Sky Quality Meter (SQM) was tested under rapidly changing temperature conditions during exposure to a stable light field in the laboratory. The reported radiance was found to be negatively correlated with temperature, but remained within 7% of the initial reported radiance over a temperature range of −15°C to 35°C, and during temperature changes of −33°C/h and +70°C/h. This is smaller than the manufacturer's quoted unit-to-unit systematic uncertainty of 10%, indicating that the temperature compensation of the SQM is adequate under expected outdoor operating conditions.

## 1. Introduction

Skyglow is the diffuse light of the sky over cities at night that occurs because artificial light emitted upward from the city scatters back towards the ground. Until very recently, skyglow had only been measured at a handful of locations around the world (e.g., [1,2]). In the last few years, however, studies of skyglow have been undertaken over large areas [3–6], and time series have been produced under different meteorological and celestial conditions [7–15].

Two of the reasons for this increased monitoring are the recognition of the biological importance of darkness, and continued growth in the amount of lighting worldwide [16]. Exposure to unnatural levels of light at night can have ecosystem level effects [17], and can alter physiological processes and hormone levels in many organisms (including humans), presumably leading to undesirable health outcomes [18]. While the strongest biological effects of artificial lighting take place directly under the lamps, the diffuse skyglow over cities may also be important [17–19]. For example, skyglow can change the behavior [20] of organisms, and can impair the navigational ability of organisms that depend upon celestial signals [19,21–23].

An additional reason for the increased monitoring is that quantitative studies of the night sky became easier to perform with the development of low-cost light meters: The “International Year of Astronomy Lightmeter” (IYAL), [10] produced by K2WLights, and the “Sky Quality Meter” (SQM), manufactured by Unihedron. Most of the studies referenced above made use of the SQM.

The response of the SQM to light was characterized by Cinzano [24] for a handheld SQM in laboratory conditions. In order to make scientific conclusions based on different skyglow meters at different locations, it is necessary to know the systematic uncertainty of the values reported by the devices. Different groups have set up experiments to track the meter-to-meter differences [25] and long term stability [11] of SQMs, but the temperature stability of the SQM has not yet been investigated.

The SQM is almost always installed and used out-of-doors, and therefore experiences fairly large temperature fluctuations. Since both ambient temperature and temperature changes can affect the response of light collecting elements and the properties of electronic components, it is prudent to check whether and to what extent SQMs are affected by temperature, and by fluctuations in temperature. In this paper, we investigate the response of two SQMs exposed to a constant light field under conditions of changing temperature in the laboratory.

## 2. Methods

### 2.1. Sky Quality Meter

The SQM measures sky radiance in the logarithmic units of mag_SQM_/arcsec^2^, which are often used by astronomers (the subscript SQM indicates that the spectral sensitivity of the SQM is not identical to the V band). The field of view (FOV) is ∼20° (full width at half maximum) for the SQM-LU (**L**ens and **U**SB readout) devices considered in this paper. To aid interpretation, all measurements have been converted to values relative to the radiance observed at the start of the measurements, using the formula 10^0.4Δ^*^m^*, where Δ*_m_* is the difference between the current and starting measurement in mag_SQM_/arcsec^2^. The starting radiance in each measurement was approximately 19 mag_SQM_/arcsec^2^, which is a typical value for the cloud-free night sky above our laboratory in Steglitz, Berlin, Germany [8].

**Figure 1.**
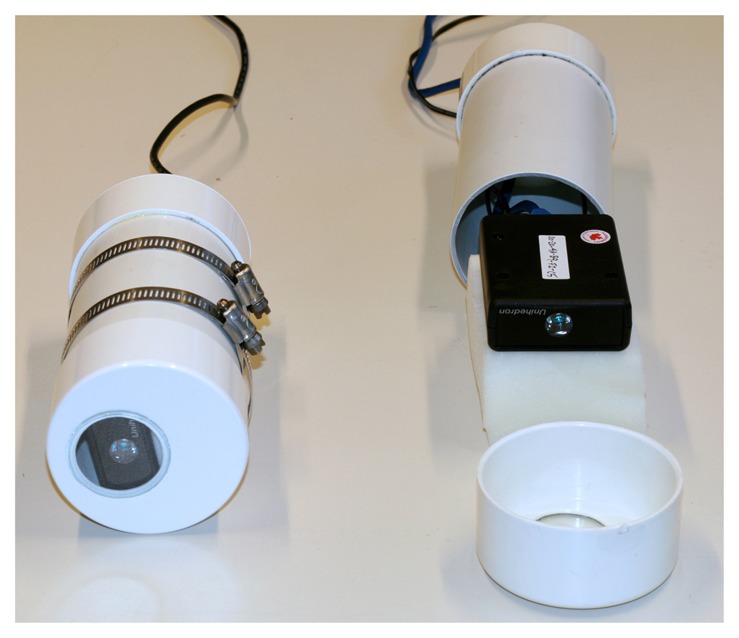
The Sky Quality Meter and the standard housing. At right is an Ethernet based SQM-LE (two cables) that is not installed inside of its housing. When the SQM is installed in the housing it appears as at left (a USB based SQM-LU with only one cable). The housing protects the SQM from the elements, and in the case the SQM-LE slightly raises the temperature relative to the surroundings. Image available under the CC BY 2.5 license. doi:10.1371/journal.pone.0017307.g002.

The SQM is not weatherproof, and its response can be affected if water enters the device (personal communication from Unihedron). It must therefore be protected from moisture by installing it in a housing, and in our experience, most users have chosen to use the housing provided by the manufacturer shown in Figure 1. The SQM housing not only protects the device from the environment but also traps the heat generated by the operation of the device. This heating is negligible in the case of SQMs connected via USB (SQM-LU), but significant in the case of Ethernet connected SQMs (SQM-LE), which tend to operate at slightly warmer temperatures. To avoid the possibility of Ethernet heating interfering with the tests, in this paper only the USB version of the SQM (SQM-LU) is studied.

The response of the light sensor of the SQM (TAOS TSL237S) has a well characterized bias with temperature, which is reported by the manufacturer. The SQM's internal software attempts to account for this bias by monitoring the temperature near the light sensor, and correcting the observed signal according to the expected bias. The temperatures that can be expected to be encountered by an SQM in the field will depend on the measurement location and time of year. In the period from 1 April 2010 to 19 August 2013 in Berlin, Steglitz, an SQM-LU mounted on the roof of our institute observed a temperature range from −16.5°C to 30.3°C during darkness. The median rate of temperature change was −0.3°C/h, and the most extreme rates of temperature change observed were −9.0°C/h and +2.6°C/h.

### 2.2. Testing Temperature Stability

**Figure 2.**
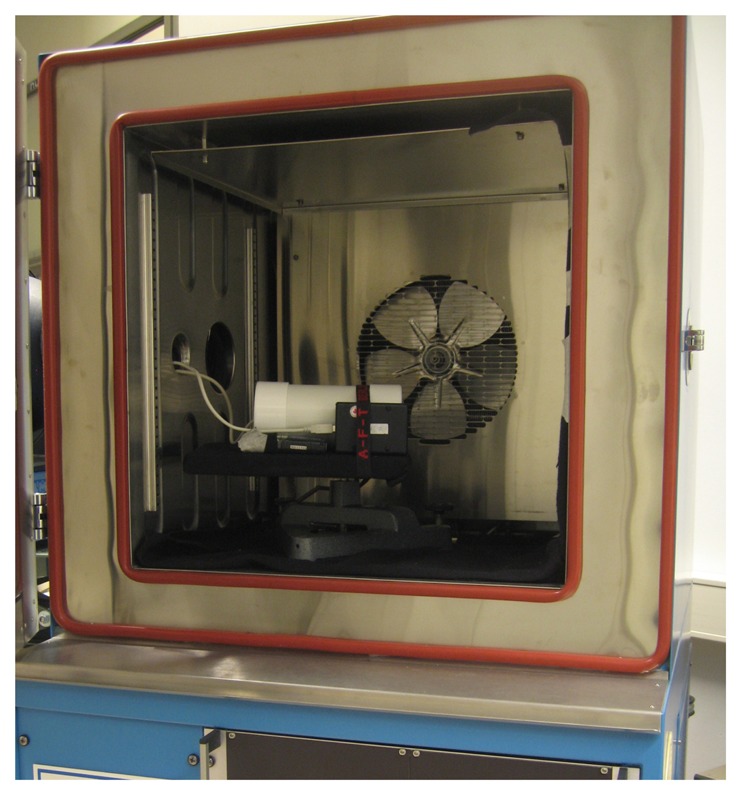
The temperature chamber used for testing the light meters is shown, with two SQM-LU devices in place. The white cylinder is the standard SQM external housing, and fixed to it is a free SQM-LU. The view direction of the SQMs is towards the right, where a dark curtain reduces the radiance to typical night sky values above our laboratory. A USB temperature logger is located behind the free SQM-LU. Light enters from a porthole on the left.

The temperature stability of the SQMs was tested by installing them inside of a programmable temperature chamber (Weiss Umwelttechnik, 125SB) designed for materials testing. Two devices were tested simultaneously, one free and one in the standard Unihedron housing as shown in Figure 2. One porthole allowed the passage of cables into the chamber, another one was left open and exposed to light from a stable, diffuse source (integrating sphere by Lichtmesstechnik Berlin). To reduce the radiance to typical values for an urban night sky, the light from the integrating sphere was incident on a dark curtain hung inside of a temperature chamber. The SQMs were manually arranged to ensure a starting radiance between 18.9 and 19.1 mag_SQM_/arcsec^2^. The stability of the light source was verified by monitoring the SQMs over a period of several days with the power to the chamber off (*i.e.*, room temperature). No variation in measured radiance was observed in either of the two SQMs.

In order to test the response of the device at different stable temperatures, the temperature chamber was programmed to run a series of temperature ramps over two days, with long pauses between the ramps (Test 1). The temperature end points and rates of change of the ramps are shown in Table 1. The device's response to extremely rapid temperature change was tested with a “stress program”, which was repeated three times (Test 2). The rates of temperature change and end points of Test 2 are shown in Table 2. The chamber's temperature was monitored using the thermometers built into the SQM-LUs, as well as a USB temperature logger (Voltcraft DL-120TH). The SQMs were read out every second, and the reported radiances were averaged over 60 s intervals.

The temperature chamber was not designed for optical measurements, and vibrated during operation. Early testing indicated that the pointing direction of the SQMs could shift during operation. The light field on the dark curtains was not perfectly uniform, so a shift in optical alignment would result in a change in observed radiance. For this reason, clamps and a heavy support plate were used to minimize this motion. The humidity of the chamber was not controlled, and dew was occasionally noted on the devices after the rapid temperature shift program completed. This could potentially have some effect on the optics, and therefore affect the observed radiance.

## 3. Results and Discussion

**Figure 3.**
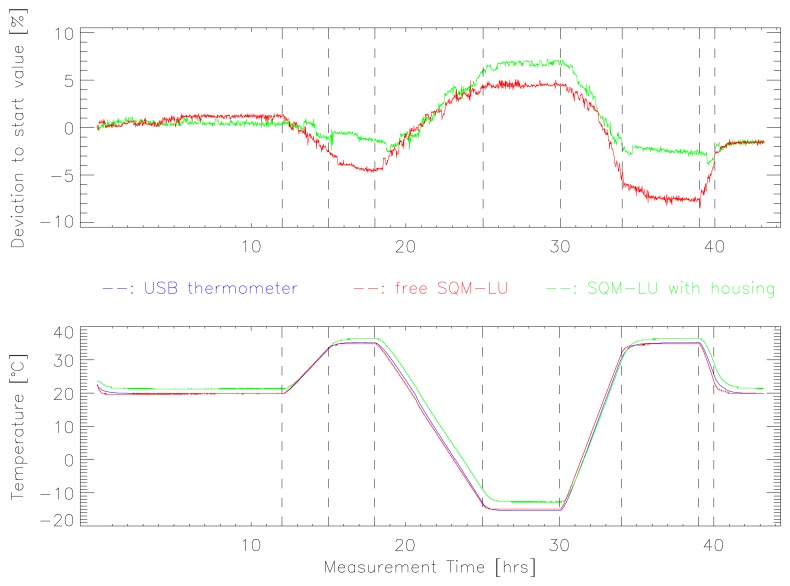
The observed deviation from the original radiance during the long temperature stability test (Test 1) is shown at top for a free SQM-LU (red), and for an SQM-LU inside of the standard weatherproof housing (green). The lower plot shows the temperature curve measured by the SQMs and the USB thermometer (blue). Dashed vertical lines show the time when temperature ramps were started or stopped.

The results of the temperature stability test (Test 1) for the two SQM-LU devices are shown in Figure 3. The upper plot shows the change in radiance relative to the initial observation over the course of the two day test for the free SQM (red) and the SQM in the housing (green). The lower plot shows the chamber temperature as measured by the SQMs and the USB temperature monitor (blue). Both SQMs had qualitatively the same deviation behavior due to rapid temperature changes. The devices reported decreased radiance at higher temperatures, and an increased radiance at lower temperatures. Once the temperature was stabilized, the reported radiance was also more stable. At the end of the test, the reported radiance for both SQMs was about 2% lower than at the start, which very likely indicates a change in optical alignment. We performed repeated measurements with the SQM inside and outside of the housing swapped, and found that the qualitative response of the SQM inside the housing was relatively consistent. The response of the free SQM was less consistent, but in no case did the reported radiance change by more than 7% relative to the starting radiance.

**Figure 4.**
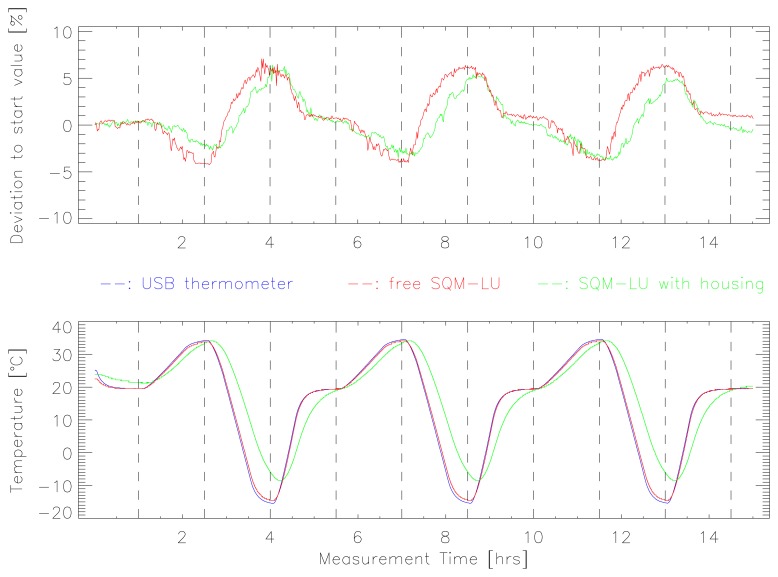
The observed deviation from the original radiance is shown for the shorter temperature stress program, which was repeated three times. The free SQM-LU is shown in red, the SQM-LU inside of the standard weatherproof housing in green. The lower graph shows the temperature curve measured by the SQMs and the USB thermometer (blue).

The data for the rapid temperature change experiment (Test 2) are shown in Figure 4. Qualitatively, the results are very similar to Test 1, and the response in each of the three cycles is very similar. The deviation in reported radiance ranged from −4% to +7% over the course of the test. At the end of the test, the devices differed by plus and minus 1% respectively, again indicating a slight change in the positioning of the SQMs.

Due to the optical limitations of the experimental setup, it is unclear whether the observed changes in radiance are due to changes in the view direction or incorrect temperature compensation of the device. Under the assumption that most of the observed change is due to a failure in temperature compensation, it is possible to derive a correction factor. The average deviation in one of the SQMs was reasonably well matched to a linear fit, while the second SQM was better described with a second degree polynomial. For the sake of simplicity, an average linear correction for all four datasets was calculated. Reported radiance could be approximately corrected using the formula:

(1) $${\text{SQM}}_{\text{corr}}={\text{SQM}}_{\text{rep}}-0.042+2.12\times 10^{-3}\times T$$

where SQM_rep_ and SQM_corr_ are the reported and corrected radiance in mag_SQM_/arcsec^2^, and *T* is the temperature reported by the SQM. Applying this correction factor to the data presented in Figures 3 and 4 reduced the worst case deviation (red curve in Figure 3) from −7% to +4% for SQM_rep_ to −5% to +1% for SQM_corr_.

With a sample of only two SQMs, it is not possible to conclude whether the correction function defined above applies equally well to all SQM devices. In addition, given that it is known that the alignment of the SQMs changed slightly during running, the correction equation provided above should be used only as an approximate guide in estimating uncertainties for measurements taken at different times with an identical meter. The uncorrected deviation reported here is small in comparison with the manufacturer's much larger quoted unit-to-unit systematic uncertainty in reported radiance of ± 10% (which we presume represents a standard deviation, and not a maximum range of differences). It is conceivable that an improved temperature compensation algorithm based on a larger scale study by the SQM manufacturer could reduce some of the measurement uncertainty.

The results of this study should be expected to apply to both the SQM-LE, as well as hand-held SQMs, which have a similar internal design. A number of groups have used an automobile in order to perform hand-held SQM measurements at widely separated distances (e.g., [4]). For the case of a heated car during the winter, very rapid temperature changes could be expected. Therefore, it is advisable that in such a measurement campaign care is taken to maintain the SQM at as close to outdoor temperature as possible while in transit between sites.

## 4. Conclusions

The stability of radiance measurements taken by two SQM-LU low light radiance meters was tested over the temperature range −15°C to 35°C, and the variation in reported radiance was found to be smaller than the 10% unit-to-unit systematic uncertainty quoted by the manufacturer. These same results can reasonably be expected to apply for SQM-LE devices as well as for hand-held SQMs. When operated in urban areas, SQMs routinely observe differences in sky radiance of an order of magnitude due to the presence or absence of clouds [8]. The temperature compensation of the SQM is therefore found to be adequate for use in the field over the range of −15°C to 35°C.

## Figures and Tables

**Table 1.** Temperature and temperature change parameters for Test 1.

**Time (h)**	0–12	12–15	15–18	18–25	25–30	30–34	34–39	39–40	40–43
Temperature (°C)	20		35		−15		35		20
Temp change (°C/h)	0	+5	0	−7.1	0	+12.5	0	−15	0

**Table 2.** Temperature and temperature change parameters for Test 2.

**Time (h)**	0–1	1–2.5	2.5–4	4–4.5
Temperature aim (°C)	20	35	−15	20
Temp change (°C/h)	0	+10	−33.3	+70
